# Establishing Natural Nootropics: Recent Molecular Enhancement Influenced by Natural Nootropic

**DOI:** 10.1155/2016/4391375

**Published:** 2016-08-30

**Authors:** Noor Azuin Suliman, Che Norma Mat Taib, Mohamad Aris Mohd Moklas, Mohd Ilham Adenan, Mohamad Taufik Hidayat Baharuldin, Rusliza Basir

**Affiliations:** ^1^Department of Human Anatomy, Faculty of Medicine and Health Sciences, Universiti Putra Malaysia, 43400 Serdang, Malaysia; ^2^Atta-ur-Rahman Institute for Natural Product Discovery, Aras 9 Bangunan FF3, UiTM Puncak Alam, Bandar Baru Puncak Alam, 42300 Selangor Darul Ehsan, Malaysia

## Abstract

Nootropics or smart drugs are well-known compounds or supplements that enhance the cognitive performance. They work by increasing the mental function such as memory, creativity, motivation, and attention. Recent researches were focused on establishing a new potential nootropic derived from synthetic and natural products. The influence of nootropic in the brain has been studied widely. The nootropic affects the brain performances through number of mechanisms or pathways, for example, dopaminergic pathway. Previous researches have reported the influence of nootropics on treating memory disorders, such as Alzheimer's, Parkinson's, and Huntington's diseases. Those disorders are observed to impair the same pathways of the nootropics. Thus, recent established nootropics are designed sensitively and effectively towards the pathways. Natural nootropics such as* Ginkgo biloba* have been widely studied to support the beneficial effects of the compounds. Present review is concentrated on the main pathways, namely, dopaminergic and cholinergic system, and the involvement of amyloid precursor protein and secondary messenger in improving the cognitive performance.

## 1. Introduction

The “nootropic” or simplified as a “smart drug,” “brain booster,” or “memory enhancing drug,” is a common term that will tag along with the compound responsible for the enhancement of mental performance. By definition, nootropic is a compound that increases mental functions including memory, motivation, concentration, and attention [1]. There are two different nootropics: synthetic, a lab created compound such as Piracetam, and notable natural and herbal nootropics, such as* Ginkgo biloba* and* Panax quinquefolius* (American Ginseng).

Natural nootropics are proven in boosting the brain function while at the same time making the brain healthier. Nootropics act as a vasodilator against the small arteries and veins in the brain [2]. Introduction of natural nootropics in the system will increase the blood circulation to the brain and at the same time provide the important nutrient and increase energy and oxygen flow to the brain [3]. Despite the 3% weight of total body weight, the brain receives around 15% of the body's total blood supply and oxygen. In fact, the brain can only generate energy from burning the glucose [4], proving that neuron depends on the continuous supply of oxygen and nutrients.

In contrast to most of other cells in the body, neuron cannot be reproduced and is irreplaceable. The neuron cells are persistently expending the converted energy to maintain the repair of the cell compartments. The energy generated from the glucose is crucial for maintenance, electrical, and neurotransmitter purposes [5]. The effect of natural nootropics is also shown to reduce the inflammation occurrence in the brain [6]. The administration of nootropics will protect the brain from toxins and minimising the effects of brain aging. Effects of natural nootropics in improving the brain function are also contributed through the stimulation of the new neuron cell. As incentive from the new neuronal cell, the activity of the brain is increased, enhancing the thinking and memory abilities, thus increasing neuroplasticity [7].

Commercialised natural nootropics in the market are reacting at different mechanisms, thus affecting different parameters. Natural nootropics alter the concentration of existing neurotransmitters. Natural nootropics have been disclosed to stimulate the release of dopamine, uptake of choline, cholinergic transmission, function of *α*-amino-3-hydroxy-5-methyl-4-isoxazole propionate (AMPA) receptor, turnover of phosphatidylinositol, and activity of phosphatase A2 [8]. Some of the natural nootropics act as a positive allosteric modulator for acetylcholine or glutamate receptor [9]. The release of neurotransmitter [10] and the increase activity of neurotransmitter [11] induced by natural nootropics facilitate the long-term potential (LTP) and improve synaptic transmission.

## 2. Suggested Molecular and Cellular Mechanisms

Establishment of new natural nootropic must consider the cellular and molecular mechanism of cognitive processes. A neuron has a known structural and functional plasticity or termed as synaptic plasticity, responsible for synaptic remodelling or known as cellular learning. Modulation in the molecular level in the neuron will alter the cognitive properties [12]. Through this review, there are few suggested mechanisms mediating the effects of nootropics in cognitive performance.

### 2.1. Glutaminergic Signalling

Glutamatergic transmission is an example of synaptic plasticity associated with LTP. Glutamate is an essential neurotransmitter involved in cognitive processes [13]. There are two different types of glutamate receptors: ionotropic (AMPA, kainite and NMDA receptors) and metabotropic receptors, distributed on the pre- and postsynaptic sites of the neuron. These receptors are responsible for neuronal network that allows the cognitive performance [14]. The release of glutamate will activate N-methyl-D-aspartate (NMDA) and AMPA receptors. AMPA receptor is responsible for synaptic transmission, while NMDA receptor is responsible for classic learning and memory [15]. The brain will respond by opening the Na^+^/K^+^ ion channel and depolarising the cell membrane [16]. However, hyperactivity of glutamate receptors can cause oxidative stress to occur responding to the cognitive dysfunction [17].

Glutamate, which acts by activating NMDA receptor, is a main excitatory neurotransmitter related in cognition function [15]. The NMDA receptor is an ionotropic channel and distributes abundantly in the hippocampus, cortex, and thalamus [18] that assists the movement of Ca^2+^, Na^+^, and K^+^ ions [19]. Activation of the NMDA receptor is reported to initiate the LTP observed in the hippocampus. LTP is part of the synaptic plasticity responsible for the physiological changes of cognitive functions [20]. LTP is remarkably studied in the hippocampus associated with learning and memory [21]. The increased Ca^2+^ permeability and blockage of voltage-dependent Mg^2+^ contribute to the synaptic plasticity and the formation of memory [19]. Increased Ca^2+^ also affects the gene and protein expression for LTP and, subsequently, may lead to neurotoxicity due to overexcitation of glutamate observed in Alzheimer's disease [16]. Blockage of NMDA receptor displays the cognitive impairment in animal models. The models are mimicking the association between the receptor to dementia [22] and schizophrenia [23] diseases. Downregulated glutamate is observed in Alzheimer's disease [24] accompanied by the reduction of NMDA receptor in the hippocampus [25].

AMPA receptor, another type of ionotropic channel, is known to mediate the fast and immediate postsynaptic response to glutamate release and thus may contribute to synaptic plasticity [26]. The receptor can be found throughout the brain, especially in the thalamus, hypothalamus, cerebral cortex, hippocampus, basal ganglia, and cerebellum [18], being also permeable for Na^+^ and K^+^ [27]. Increased density of AMPA receptor in the hippocampus was shown to enhance the memory consolidation [28]. The use of AMPA modulator causes the deactivation and desensitisation of the receptor in the hippocampus thus subsequently facilitating the cognitive performances, including short-term memory [29].

### 2.2. Cholinergic System

In regulating the cognitive functions, the central cholinergic system is suggested to be an essential neurotransmitter associated with, namely, acetylcholine (ACh) [30]. The neuronal nicotinic ACh receptor is established located on the presynaptic terminal and applies an action on hippocampal synaptic transmission via stimulating the release of glutamate [31]. The activation of nicotinic ACh receptor is stimulated by activation of protein kinase C (PKC). These events subsequently maintain the phosphorylation of the receptor [32] and sustain the upregulation of glutamate release. Afterward, the high expression of glutamate initiates the long-lasting acceleration of hippocampal synaptic transmission [33]. Taking piracetam as an example, nootropics are suggested to involve the biochemical modifications in the aged brain [34]. Treatment of nootropics shows pronounced effects in the impaired brain functions induced by number of noxious stimuli, for example, hypoxia, aging, and injury [35].

Cognitive dysfunction is related to diminish cholinergic function, treated by stimulation of central cholinergic activity expressing the improvement of cognitive performances [36]. The loss of neuronal cholinergic observed in the hippocampal area is responsible for the major characteristic of Alzheimer's disease. In treating Alzheimer's disease-type senile dementia, central cholinergic system is suggested to be improved. Administration of established nootropics is established to increase the level of ACh and upregulation of receptor binding for cholinergic in the frontal cortex and hippocampus [37]. Downregulation of noradrenergic function is studied to diminish the behavioural impairment due to degeneration of cholinergic system [38]. The nootropics activities are observed through the downregulated ACh esterase activity. The reduction subsequently leads to upregulation of ACh expression in the brain. Thus, good agents of nootropics are able to decrease norepinephrine (NE) and elevate the 5-hydroxytryptamine (5-HT) expression observed in the central cortex, hippocampus, and hypothalamus [39].

### 2.3. Amyloid Precursor Protein

Dysfunction of the cholinergic system in Alzheimer's disease is also accompanied by the involvement of amyloid protein, specifically amyloid *β*-protein, and neurofibrillary tangles [40]. Modulation of processing of cellular component is also influenced by the neuronal transmission and synaptic plasticity. Amyloid precursor protein (APP) is one example of cellular component affected. APP is detected in the membrane of synaptic preparation and leading to the involvement of a fragment of APP in synaptic formation and maintenance [41]. The consequences of the influence of APP seem to contribute to the memory formation [42]. Introduction of natural nootropics increases the learning and memory performance, which causes upregulation of APP expression [43]. Knockout APP in mice was observed to impair the behavioural activity and alter the structure and length of the neuron [44]. In introducing natural nootropic, modulation of APP processing must be approached since it mediates the formation of specific neurotropic APP fragments, which is important for memory functions.

Patients diagnosed with Alzheimer's disease are expressed with the deposition of insoluble or oxidised amyloid-*β* derived from APP present in the brain [45]. *β*-amyloid peptide is another fragment derived from the APP, contributing to the impairment of short-term working memory [46]. Oversynthesis of *β*-amyloid from APP may be influenced by the increased neuronal activity thus subsequently causing the depression of synaptic transmission [47]. The patient's brain also contains an activated caspase-3. Caspase-3 is a cysteine protease that facilitates the apoptosis induced by the mitochondrion [48]. Marx [49] has listed a possible reason of the onset of Alzheimer's disease, namely, due to deposition of amyloid-*β*, apoptosis, and presence of oxidative stress. The amyloid-*β*-induced apoptosis leads to the neuronal degeneration [50]. High expression of amyloid-*β* in the neuron stimulates neuronal apoptosis death due to induction of caspase-3 activities [51]. Production of amyloid-*β* fibril is an indicator for development of Alzheimer's disease. The amyloid-*β* fibril is responsible for permeability of lipid membrane [52] and stimulation of Ca^2+^ conductance [53].

### 2.4. Secondary Messenger

Schwartz [54] has claimed the involvement of secondary messenger implicated in the cognitive purpose. The evolution of the intracellular signalling cascade involves various enzymes and selective protein-protein interactions in response to the cognitive performance. LTP, as mentioned before, is related to the activation of NMDA receptor and leads to influx of Ca^2+^. It is originating the series of events causing the activation of pre- and postsynaptic mechanisms [55]. Ca^2+^ is observed to activate PKC in the dentate gyrus [56], a molecule that is involved in learning and memory processes [57]. The administration of PKC activator [58] and nootropic drugs were observed to improve the memory performances, suggesting the involvement of similar pathway, the PKC pathway [59].

Upon PKC activation, it localises to specific subcellular sites and confers different physiological function [60]. The failure for this translocation to occur is found in normal ageing and number of neuronal pathologies [61]. Considering the role of PKC in learning and memory mechanisms, PKC improves synaptic plasticity in the brain. In addition, diminished calcium/calmodulin-dependent protein kinase II (CaMKII) and PKC activity contribute to downregulation of NMDA receptor, thus reducing the release of glutamate [62].

### 2.5. Miscellaneous

Insulin receptor becomes another target for investigating the effect of nootropic in cognitive purposes. Insulin plays a role in the neuropathological views, including involvement in neurotropic and neuromodulatory functions [63]. In the central nervous system (CNS), insulin is synthesised and released from neuron as a response to depolarisation [64]. As observed from a number of studies, insulin receptors are also responsible for learning and memory [65]. High expression of insulin receptors is found in the hippocampus, including in the dentate gyrus and CA1 pyramidal cells [66]. Neuron insulin or insulin receptor in the hippocampus can modulate the synaptic activity mediated by NMDA, which subsequently suppress the AMPA and GABA_A_ receptors. It alters the synthesis and the activity of a number of neurotransmitters [67]. Activation of neuronal insulin will stimulate the signalling pathway of cognitive function, including activation of mitogen-activated protein kinase (MAPK), PKC, and phosphatidylinositol-3-kinase (PI3K) [67]. Diabetic rats were observed to experience cognitive impairment and LTP [68].

Other than insulin receptor, angiotensin receptor also facilitates the signal transduction in the CNS. Angiotensin, a peptide hormone, is part of the renin-angiotensin system that stimulates the release of aldosterone from the adrenal cortex. Angiotensin II (Ang II), one of the subtypes of angiotensin, regulates the blood pressure via a number of actions. The most significant actions are vasoconstriction, renal actions, and increased aldosterone biosynthesis [69]. Ang II also interacts with other neurotransmitters, causing the release of noradrenaline and the synthesis of serotonin [70]. Ang II facilitates the cognitive and behavioural processes through its specific receptors and metabolites expressed in animal models [71]. Upon cognitive impairment of Alzheimer's disease patient, the Ang II in the brain inhibits the release of acetylcholine through inhibition on angiotensin type 1 receptor (AT1) [72]. Acetylcholine is known as a critical neurotransmitter responsible for memory.

Review by van der Staay et al. [73] has established a definition of cognition enhancer. Overall, a cognition enhancer is a pharmacological compound that enhances the mnemonic and cognitive function that can cross the blood-brain barrier. The cognition function is influenced by the cognition enhancer including the learning, consolidation and retrieval, and memory. As a contrast, it does not have psychopharmacological activity such as sedation and has minimal or no adverse effects with low toxicity level. Listed below are examples of natural nootropic that were proved to improve cognitive and memory properties.

## 3. Examples of Nootropic

### 3.1. Pyrrolidinone Derivatives

Pyrrolidinone is a class of 5-membered lactams with a four-carbon heterocyclic ring structure with biological interest [74] found in many pharmaceuticals and natural products. The synthesis of nootropic from pyrrolidinone derivatives has common features including enhancing the learning process, diminishing the impaired cognition, and protecting against brain damage. Number of pyrrolidine derivatives are commercially available, including piracetam, oxiracetam, aniracetam, and promiracetam [75]. Administration of aniracetam or piracetam affects the muscarinic receptor binding in the different brain regions [76]. Study by Pilch and Müller [77] had established the upregulation of m-cholinoceptor in the brain responding to the aging brain.

Piracetam or 2-oxo-1-pyrrolidineacetamide, a cyclic derivative of gamma-aminobutyric acid (GABA) [78], is widely used in treating senile dementia and Alzheimer's disease [79]. Studies showed the role of piracetam in enhancing the memory and learning [80] and act synergistically with choline leading to greater enhancement of cognition. Winnicka et al.'s study [78] showed the effect of piracetam on regulating the release of glutamate observed in the cortex and hippocampus, suggesting involvement of NMDA receptor induced by piracetam. Despite having a low affinity for glutamate receptors, piracetam initiates a number of effects on the receptors, for example, on AMPA receptor. Treatment of piracetam causes activation of AMPA receptor thus stimulating the influx of Ca^2+^ in the brain and increasing the density of AMPA receptors in the synaptic membrane of the cortex. Piracetam also causes the release of glutamate stimulated by potassium in the hippocampal nerves [81]. Recent reports suggest the neuroenhancing effect of piracetam is via stimulation of acetylcholinergic and glutamatergic systems, plus elevation of membrane permeability [82].

Introduction of aniracetam is usually related to the involvement of AMPA receptor [83], cholinergic system [84], and metabotropic receptor [85], as part of cognition function. Aniracetam, including pyrrolidinone derivative compounds, is established to diminish the cognitive impairment [86]. Systemic administration of aniracetam improves the cognitive performance observed behaviours, suggesting the involvement of AMPA in the dentate gyrus [87]. The effects of cognitive enhancer of aniracetam are postulated due to the slow rate of deactivation [88] and desensitisation of AMPA receptors [89] observed using hippocampal slide. Other studies had suggested the involvement of activated hippocampal PKC and sustained ratio of membrane and cytosolic PKC*γ* [90]. The enhancement of PKC*γ* is subsequently induced by the phosphorylation of glutamate receptor subunits, thus modifying the channel kinetics of AMPA receptor [91]. The study showed that the intrahippocampal aniracetam mediates the formation of behavioural LTP, thus representing the synaptic mechanism induced by the treatment [83].

Another example of pyrrolidinone derivative is nefiracetam (N-(2,6-dimethyl-phenyl)-2(2-oxo-1-pyrrolidinyl)), a piracetam-like nootropic agent. Studies show that the compound improves the impaired cognitive due to drugs [92], morphine [93], or ageing [94]. Intake of nefiracetam is postulating the cholinergic system, as ACh receptor enhances the release of neurotransmitter [31]. Nefiracetam is studied to influence the phosphorylation of nicotinic ACh receptor by activating the PKC, thus helping the release of neurotransmitter from the presynaptic terminal [32]. Synaptic transmission influenced by nefiracetam is not mediated through the blocking of GABAergic transmission and enhanced postsynaptic ionotropic glutamate receptor. Interestingly, nefiracetam is improving the synaptic strength by aiming at the nicotinic ACh receptor [33] possibly by Na^+^ without affecting the permeability of Ca^2+^ [32]. Another study suggests that the inhibition of PKA is responsible for the effect of nefiracetam on Ca^2+^ channel [32]. In contrast to piracetam and oxiracetam, nefiracetam enhances the N- and L-type Ca^2+^ channels but not T-type [95].

DM235 or sunifiram is a recent compound structurally related to piracetam and is known to prevent cognitive deficits. The compound is observed to improve the impaired cognitive functions by inhibiting the induction of amnesia [96]. As discussed previously, pyrrolidinone derivatives prevent the amnesia induced by the impaired cholinergic system [97] and ameliorate the cognitive deficits [98]. Similar to other pyrrolidinone derivatives, sunifiram increases the release of neurotransmitter from the presynaptic terminal [99]. Amnesia can be induced by altering the neurotransmitter system through GABA. Activation of GABA receptor impairs the cognitive function including the learning and memory processes [100]. In contrast to other pyrrolidinone derivatives, sunifiram is more potent while having a similar characteristics with piracetam. Sunifiram is observed to ameliorate the memory function and has less adverse effects [81]. A recent study reports the improvement of hippocampal LTP induced by sunifiram is mediated by the glycine-binding site of NMDA receptor [101], an attractive binding site for Alzheimer's disease drugs [102]. Thus, the reaction of sunifiram on the same binding site is suggested to ameliorate the impaired cognitive function of Alzheimer's disease patients [101]. Stimulation of the binding is also associated with the increased autophosphorylated CaMKII and PKC*α* thus suggesting the enhanced memory and hippocampal LTP [103].

### 3.2. *Bacopa monnieri*

**Figure 1:**
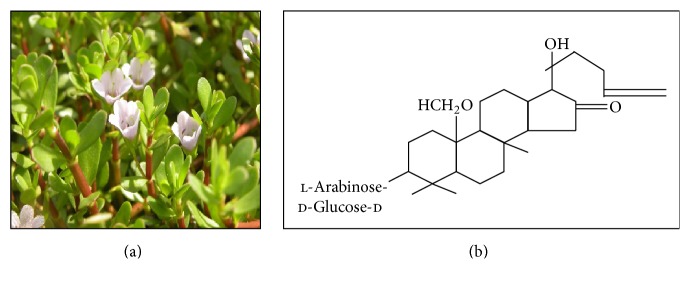
*Bacopa monnieri*. The plant has purple flowers with oblong leaves found throughout the Indian subcontinent. This plant is classified under the family of Scrophulariaceae. On the right is the chemical structure for bacosides [107].

*Bacopa monnieri* or Brahmi is derived from the family of Scrophulariaceae, found throughout the Indian subcontinent in a wet, damp, and marshy area [104]. It has purple flowers with numerous branches and small oblong leaves (Figure 1). This plant is known to be used for number of nervous system disorders, including insomnia, anxiety, and epilepsy. According to Ayurvedic medical practitioners,* Bacopa monnieri* is categorised as a medhya rasayana, a compound that stimulates and enhances the memory and intellect. These properties were studied preclinically and clinically [105]. The property of memory, facilitating action of this plant, is contributed by the chemical constituents of bacoside A, assigned as 3-(a-l-arabinopyranosyl)-O-*β*-d-glucopyranoside-10, 20-dihydroxy-16-keto-dammar-24-ene [106], and bacoside B [107]. The treatment of a mixture of bacosides A and B is mediating the three types of learning function, procedural, declarative, and spontaneous, and improves the episodic memory observed in animal models [108]. Beside enhancing cognition and memory functions,* Bacopa monnieri* are also known for their anxiolytic effects and in managing the convulsive sicknesses [109].

Singh and colleagues [110] had suggested the membrane dephosphorylation triggered by bacosides concurrently leads to elevation in protein and RNA turnover observed in certain brain regions. The nootropics effect of* Bacopa monnieri* is mediated by enhancement of protein kinase activity and production of protein in the hippocampus [39]. Study done by Anand and colleagues [111] demonstrated the characteristics of natural antioxidant and DNA damage preventing agent of the* Bacopa monnieri*. Other effects of* Bacopa monnieri* are including hepatoprotective agent against morphine toxicity [112], calcium antagonist [113], anticancer agent [114], and antiaddictive agent [115]. Despite that, the combination of bacosides A and B also was studied to express antistress property [116], protecting the brain from smoking induced membrane damage [117], and protective function against d-galactosamine induced liver injury [118].

### 3.3. Nicotine

**Figure 2:**
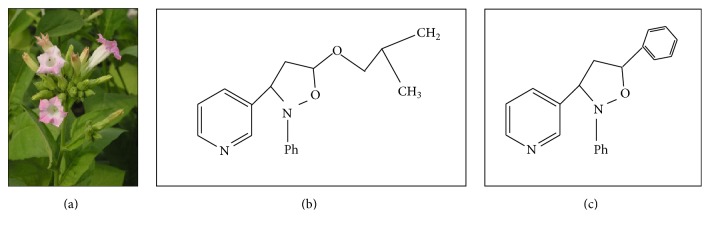
*Nicotiana mutabilis*. The plant is classified under the family of Solanaceae and contains nicotine as psychoactive compound (a). Compound A, syn-5-isobutoxy-2-phenyl-3-(3-pyridyl)-isoxazolidine (b), and compound B, syn-2,5-diphenyl-3-(3-pyridyl)-isoxazolidine (c), are example of synthesised nootropics derived from nicotine for learning and memory purposes [122].

Nicotine is a potent parasympathomimetic alkaloid derived from the family of plants of Solanaceae (Figure 2). The psychoactive nicotine is found in the leaves of* Nicotiana rustica*, the tobacco plant* Nicotiana tabacum*,* Duboisia hopwoodii*, and* Asclepias syriaca* [119]. Despite its addiction liability and undesired adverse effects [120], nicotine is found to improve learning and memory properties and enhance the memory impairment due to lesion of the septohippocampal pathways or aging. Downregulated expression of nicotinic receptor is observed in Alzheimer's disease patients [121].

Due to the prohibition of the use of nicotine, novel nicotine analogue is synthesised and evaluated, namely, syn-5-isobutoxy-2-phenyl-3-(3-pyridyl)-isoxazolidine (compound A) and syn-2,5-diphenyl-3-(3-pyridyl)-isoxazolidine (compound B) [122]. Nicotine and its synthesised analogs are established to react on different pathways, expressing improvement in memory [123]. Compounds A and B are postulated to stimulate the release of acetylcholine through the activation of presynaptic nicotine acetylcholine receptors. These receptors are responsible for modulating the release of neurotransmitter [124].

### 3.4. *Ginkgo biloba*

*Ginkgo biloba* or maidenhair tree is the only species derived from family of Ginkgophyta and the order of Ginkgoales. It is called a “living fossil” since the morphology and features of the plant are changed for over 100 million years [125]. The plant is well-known for its medical used as well as being a source of food [126]. Despite the lack of reports,* Ginkgo biloba* is claimed to have neuroprotective effects observed in human and animal models [127]. A recent report has suggested the effect of* Ginkgo biloba* in treating Alzheimer's disease patient or other cognitive disoders.* Ginkgo biloba* also has been listed under group of antidementia drugs [128]. It acts as antioxidant and antiapoptotic properties and also induces inhibition effects against caspase-3 activation and amyloid-*β*-aggregation toward Alzheimer's disease.

**Figure 3:**
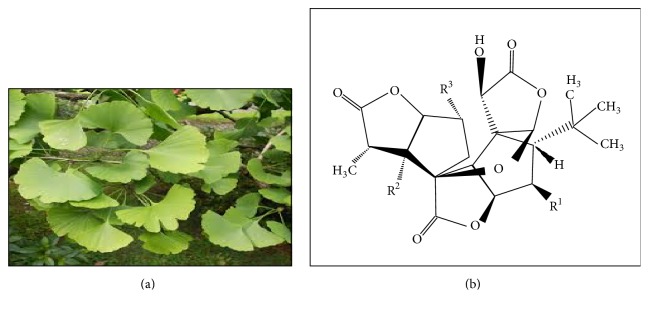
*Ginkgo biloba*. The plant is classified under the family of Ginkgoaceae, the only species in the division of Ginkgophyta. With 40 m in height, this tree is characterised by the fan-shaped leaves composed of more than two distinct lobes (a). Ginkgolide A, R^1^=H; R^2^=H; R^3^=OH, Ginkgolide B, R^1^=OH; R^2^=H; R^3^=OH, Ginkgolide C, R^1^=OH; R^2^=OH; R^3^=OH, Ginkgolide J, R^1^=H; R^2^=OH; R^3^=OH, Ginkgolide M, R^1^=OH; R^2^=OH; R^3^=H (b) [129].

The extract of the leaves diminishes the amyloid-*β* fibrillogenesis, reduces the apoptosis induced by mitochondria, and downregulates the caspase-3 activity [51]. This plant also is proposed to have antiamyloidogenic property whereby the plant extract prevents the production of amyloid fibrils [51]. The compound found in* Ginkgo biloba*, terpenoid, namely, bilobalide and ginkgolide, is observed to be involved in the caspase-3 activation [51]. Nakanishi [125] has proposed the memory enhancing effect of ginkgolides. The ginkgolides are compounds in the plant that terminate the effects of amyloid peptide on LTP (Figure 3).

### 3.5. *Panax ginseng*

*Panax ginseng* (Asian ginseng) is described as the “king herb" and has an important position in the traditional Chinese medicine [130]. A lot of reports are discussing the role of* P. ginseng* especially in improving the cognition function of Alzheimer's disease patients. Antioxidant property in* P. ginseng* is claimed to suppress Alzheimer's disease-like pathology [131]. The intake of* P. ginseng* in healthy individuals is observed to increase the memory performances [132].

**Figure 4:**
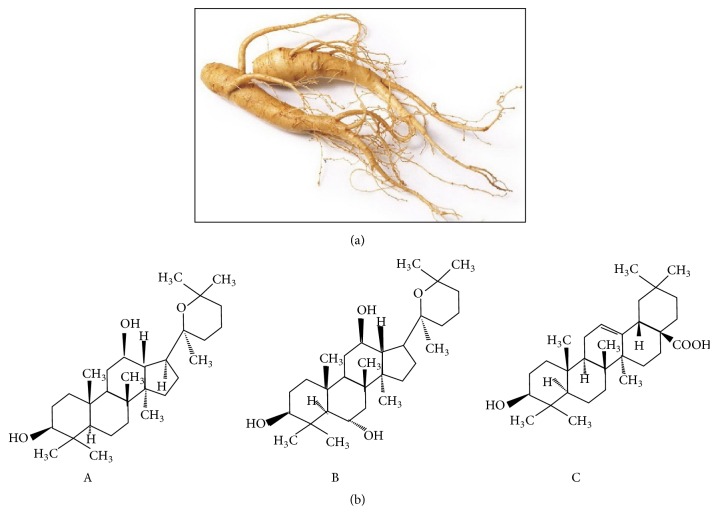
*Panax ginseng*. Ginseng belongs to the genus* Panax* of the family Araliaceae, found in the cooler climates. The name of the plant is derived from the Chinese term meaning “person” and “plant root” due to the feature of the root that resembles the legs of a person (a). Ginsenosides, the principal bioactive compounds of* P. ginseng*. A, Panaxadiol; B, Panaxatriol; C, oleanolic acid (b) [137].

The active constituents of the* Panax* spp. are ginsenoside saponins, which are divided into Panaxadiol, Panaxatriol, and oleanolic acid groups. The Panaxadiol and Panaxatriol groups are studied to increase the release of neurotransmitters in the brain [133]. Other ginsenosides affect the secretion of corticosterone and uptake of NE, dopamine, serotonin, and GABA [134]. It is suggested that the high ratio of Panaxatriol to Panaxadiol is responsible for the enhancement of memory and cognitive properties [135].* P. quinquefolius* (American ginseng) has a lower ratio of Panaxatriol to Panaxadiol as compared to* P. ginseng* (Asian ginseng) (Figure 4) [136].

### 3.6. *Rhodiola rosea*

**Figure 5:**
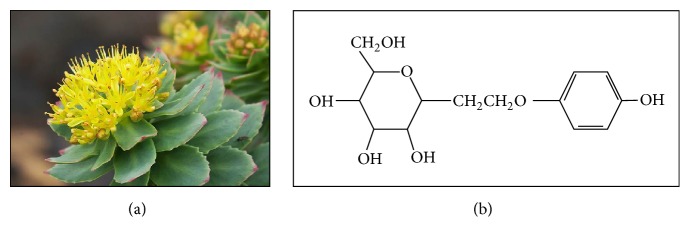
*Rhodiola rosea*. It belongs to the family of Crassulaceae.* R. rosea* is growing on the sea cliffs and on the mountains. The plant is dioecious, with yellow to greenish yellow flowers (a). Salidroside is claimed as an active constituent responsible for neuroprotective and antioxidant properties (b) [140].

*Rhodiola rosea *(*R. rosea*), known as golden root and Arctic root, is reported to improve cognitive function [138], enhance memory and learning [139], and protect the brain [140]. Belonging to the family of Crassulaceae, this plant is observed to increase the level of 5-HT and NE in the cerebral, prefrontal, and frontal cortex [139]. At the same time, the intake of* R. rosea* causes the upregulation of DA and ACh in the limbic system pathways, responsible for emotional calming [141], as* R. rosea* is acting as antioxidant agent. The study showed that the introduction of* R. rosea* may protect the nervous system against oxidative damage, thus lowering the risk of Alzheimer's disease onset. The treatment of the plant also enhances the learning and memory impairment in Alzheimer's disease [142]. Sharing the same property with* Bacopa monnieri* and* Panax ginseng*,* R. rosea* is considered to be an “adaptogen” that enhances endurance, resistance, and protest against stressful situation [143]. Salidroside, an active component of* R. rosea*, is claimed to have neuroprotective and antioxidative effects (Figure 5) [140].

## 4. Conclusion

The understanding of the mechanisms influenced by the administration of natural nootropics has been expanded tremendously in this past decade. Establishing natural nootropic is challenging as optimum dose has to pass blood brain barrier so that it can stimulate responding mechanism. In the same time, the nootropic is helping the body systems such as blood circulation as well as energy booster. There are a number of mechanisms influenced by the administration of nootropics, such as glutaminergic signalling and amyloid precursor protein, also responsible for neuro-related diseases such as dementia and Alzheimer's disease. Thus, the understanding of the mechanism stimulated by nootropic is expected to increase the cognitive performances of the cognitive impairment patients.
